# Lacosamide-Related Arrhythmias: A Systematic Analysis and Review of the Literature

**DOI:** 10.7759/cureus.20736

**Published:** 2021-12-27

**Authors:** Ruchi Yadav, Ezra Schrem, Vivek Yadav, Amog Jayarangaiah, Sushruth Das, Pramod Theetha Kariyanna

**Affiliations:** 1 Internal Medicine, Brookdale University Hospital and Medical Center, New York City, USA; 2 Internal Medicine, State University of New York Downstate Health Sciences University, New York City, USA; 3 Pulmonary and Critical Care, State University of New York Downstate Health Sciences University, New York City, USA; 4 Internal Medicine, Marshfield Clinic Health System, Marshfield, USA; 5 Internal Medicine, Trinity School of Medicine, Kingstown, VCT; 6 Interventional Cardiology, Marshfield Clinic Medical Center, Eau Claire, USA

**Keywords:** echocardiography, electrocardiography, sinus pauses, av block, heart block, atrial fibrillation, ventricular tachycardia, arrhythmias, : lacosamide

## Abstract

Lacosamide (LCM) is a new antiepileptic drug used as an adjunctive treatment for partial seizures with and without secondary generalization. One of the modes of action is the enhancement of slow inactivation of voltage-gated sodium channels. Experimental studies and clinical trials suggest that LCM acts upon both neurons and the heart and may increase the risk of cardiac arrhythmias. A systematic review was conducted to investigate characteristics of arrhythmias related to the use of LCM for the treatment of seizures. The search terms “lacosamide”, “arrhythmias”, “AV block”, “atrial fibrillations/flutter”, “cardiac conductions defects”, “ventricular tachycardia”, “ventricular fibrillation were used. Case reports and retrospective studies were gathered by searching Medline/PubMed, Google Scholar, CINAHL (Cumulative Index to Nursing and Allied Health Literature), Cochrane CENTRAL (Cochrane Central Register of Controlled Trials), and Web of Science databases. Seventeen articles were selected for review. Ventricular tachycardia was the most reported LCM related arrhythmia (29.4%), followed by new-onset atrial fibrillation (17.6%), complete heart block (17.6%), Mobitz type 1 Atrio-ventricular block (11.8%), sinus pauses (11.8%), pulseless electrical activity (5.9%) and widening QRS complex (5.9%). Further research and clinical trials are needed to explore the etiopathogenesis and causative relationship between the use of LCM and arrhythmias.

## Introduction and background

Lacosamide (LCM) is a new antiepileptic drug approved by the United States Food and Drug Administration (FDA) in October 2008 as an adjunctive treatment for partial seizures with and without secondary generalization [1]. It is composed of (R)-2-acetamido-N-benzyl-3-methoxyproionamide, causing slow inactivation of voltage-gated sodium channels in neurons [2]. Based on the above mechanism of action several reports of dose-dependent cardiac arrhythmias have been reported in the literature [3-6]. LCM inhibits cardiac sodium channel SCN5A that could be an underlying possible mechanism for cardiac arrhythmias, including ventricular tachycardia, sinus pauses, atrial fibrillation, and sudden death [7, 8]. Limited data is available on the relationship between the use of antiepileptic drugs/LCM and cardiac arrhythmias. Multiple isolated cases of arrhythmias have been associated with LCM use [5, 6, 9]. Here we present a systematic review of such cases of LCM-related arrhythmias to evaluate the need for assessment of risk factors and potentially warn physicians of the dose-related cardiac effects of LCM.

## Review

Method

**Figure 1:**
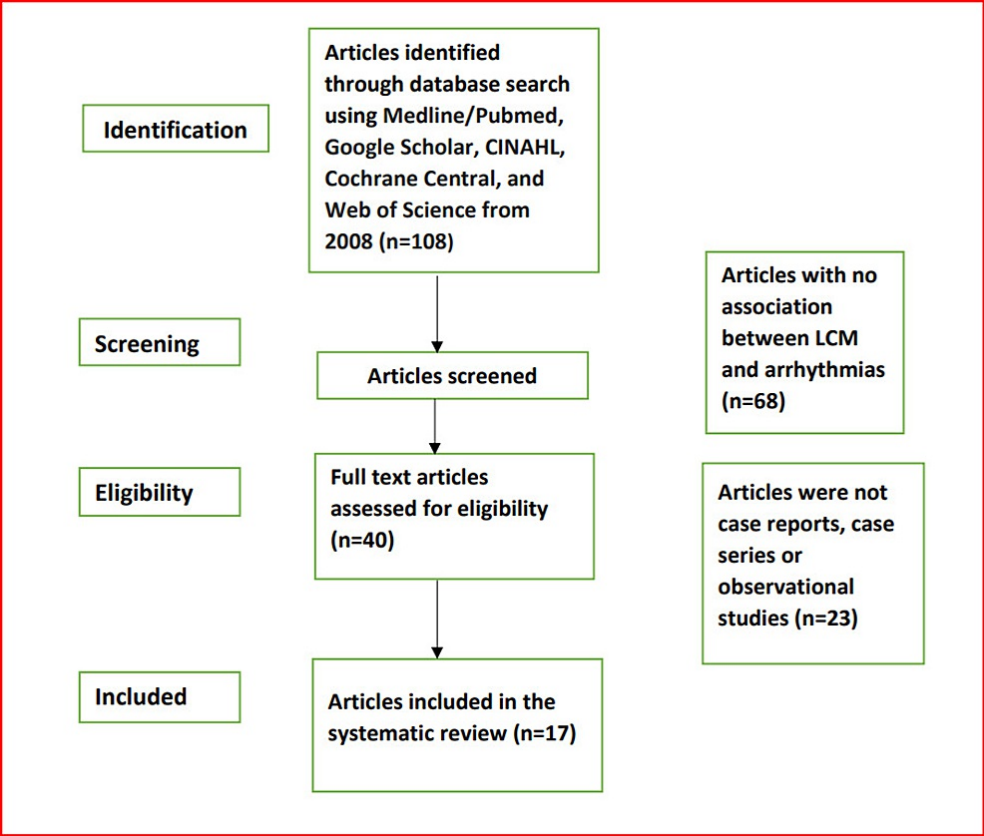
Flow diagram of literature search and selection criteria adapted from the Preferred Reporting Items for Systematic Reviews and Meta-Analyses (PRISMA) LCM-lacosamide

A comprehensive literature search was conducted by two authors, using Medline/Pubmed, Google Scholar, CINAHL (Cumulative Index to Nursing and Allied Health Literature), Cochrane CENTRAL (Cochrane Central Register of Controlled Trials), and Web of Science databases, for relevant studies since 2008. The terms “lacosamide, arrhythmias, AV block, atrial fibrillations/flutter, cardiac conductions defects, ventricular tachycardia, ventricular fibrillation, cardiac conductions defects” were used to identify cases of myocardial arrhythmias associated with LCM use. A total of 108 articles were found related to LCM and arrhythmias. Only articles that reported LCM use and the presence of cardiac arrhythmias were included. Seventeen studies that included the case reports were then deemed eligible for inclusion in this review as shown in Table 1. Studies were excluded if: 1) Articles were not case reports, case series or observational studies, or 2) Articles were reviews or editorials. The reference list of each report was reviewed for potential additional cases. All cases were reviewed in detail. The present analysis was performed according to the Preferred Reporting Items for Systematic Reviews and Meta-Analyses (PRISMA). A PRISMA flow diagram detailing the process of identification, selection, and inclusion of studies is shown in Figure 1.

**Table 1:** Summary of study characteristics of all the searched articles. ACLS-advanced cardiac life support; Afib-atrial fibrillation;  Aflutter-atrial flutter;  AR-aortic regurgitation;  AV-atrioventricular;  AVB-atrioventricular block;  AVR-aortic valve repair;  bpm-beats per minute;  CAD-coronary artery disease;  CCU-coronary care unit;  CHF-congestive heart failure;  CVA-cerebrovascular accident;  CV-cardiovascular;  dced-discontinued;  EF-ejection fraction;  EKG-electrocardiography;  f.b.-followed by;  F-female;  G1DD-grade1 diastolic dysfunction;  HLD-hyperlipidemia;  HOCM-hypertrophic obstructive cardiomyopathy;  HR-heart rate;  HTN-hypertension;  ICH-intracranial hemorrhage;  IVF-intravenous fluids;  JER-junctional escape rhythm;  LCM-lacosamide;  LFB-left fascicular block;  LVH-left ventricular hypertrophy;  MAT-multifocal atrial tachycardia;  M-male;  MR-mitral regurgitation;  MVR-mitral valve replacement;  NA-not available;  NCT-narrow complex tachycardia;  NE-norepinephrine;  NSR-normal sinus rhythm;  PEA-pulseless electrical activity;  PHT-pulmonary hypertension;  PVC-premature ventricular complexes;  RAD-right axis deviation;  RBBB-right bundle branch block;  RHD-rheumatic heart disease;  RVR-rapid ventricular rate;  SB-sinus bradycardia;  ST-sinus tachycardia;  TCP-transcutaneous pacing;  TIA-transient ischemic attack;  TR-tricuspid regurgitation;  TTE-transthoracic echocardiography;  VF-ventricular fibrillation;  VT-ventricular tachycardia;  WCT-wide complex tachycardia;  WMA-wall motion abnormalities;  WNL-within normal limits

Year of publication, author[references]	Age/sex	CV risk factors	Indication for LCM	EKG	TTE	Arrhythmia	Final diagnosis of arrhythmia	Mx of arrhythmia	Cardiac arrest	Death
2010, Digeorgia [1]	37/F	None	Seizures	Aflutter, HR~100-136 bpm, turning into afib	NA	New-onset aflutter/afib	Afib	LCM dced	No	No
2011, Nizam [5]	45/M	None	Seizures	Mobitz 1 & RBBB	WNL	Mobitz type 1 & bradycardia	Mobitz type I	LCM dced	No	No
2011, Krause [6]	89/F	HTN, Hypokalemia	Seizures	Complete AV nodal block 15 mins after LCM bolus, 30 mins later 1^O^ AVB, NSR later	NA	Complete heart block and pauses x3 up to 10 secs	AVB type III	LCM dced and IVF resuscitation	Yes	Yes
2011, Wittstock [10]	81/F	HTN, CAD, 1^O^ AVB, left cerebellar CVA, hypokalemia	Seizures	Initially 1^o^ AVB, after LCM complete AVN block, later on, aflutter	NA	Complete heart block with asystole for 30 sec	AVB type III	Atropine, LCM dced & metoprolol	No	No
2012, Kaufman [11]	67/F	Hypokalemia	Seizures	Initial NSR~ 96 bpm with PVCs, after LCM Afib with RVR~ 132 bpm	No WMA, EF~55%, Mild AR, TR, mod PHT	New-onset Afib with RVR~132 bpm	Afib	Metoprolol 2.5 mg IV x1, f.b. spontaneous resolution after 8h, LCM dced	No	No
2013, Digeorgia [7]	49/M	HTN, HLD, low HR variability	Seizures	1^O^ AVB with LFB & severe QRS widening, NSR after LCM was dced	NA	Sustained VT on outpatient stress test	VT	LCM dced	No	No
2013, Chinnasami [12]	49/F	None	Seizures	Baseline before LCM- SB~54 bpm After LCM- interval sinus pauses with JER	NA	JER & pauses (33 times, longest~ 6.24 sec), also ST~118 bpm & SB~36 bpm	Sinus pause	Holter monitor and LCM dced	No	No
2015, Loomba [13]	3/M	Congenital hypoplastic left heart syndrome, well-controlled MAT	Seizures	Baseline-NSR, wide complex tachycardia~ 240-260 bpm after use of LCM, NSR-post-discharge	NA	WCT~240-260 bpm, in addition to NCT with 1:1 and 2:1 ventricular response & gradual complex widening during non- sustained runs	VT	Amiodarone infusion, flecainide toxicity treatment (bicarb, intralipids, isoproterenol)- not available hence eventual cardioversion	No	No
2015, Chua-Tuan [14]	16/F	None	Overdose toxicity	Admission-ST@139 bpm, then ST~108BPM terminal RAD	WNL	Cardiac arrest, secondary to pulseless VT with bradycardia & asystole	VT	ACLS protocol with shocks, epinephrine, atropine, bicarbonate, on NE drip	Yes	No
2017, Berei [15]	70/F	HTN, RHD s/p AVR and MVR, Type 2 ischemic pain	Seizures	Baseline EKG~NSR After LCM Sinus node dysfunction with LBBB and widened QRS~160 msec	AR due to malfunctioning AV bioprosthesis.EF 65%	Wide complex monomorphic VT 2 hours after second dose of LCM	VT	Amiodarone infusion, cardioversion LCM dced	No	No
2018, Lachuer [16]	88/F	HTN, angina	Seizures	NSR and LBBB at baseline. After LCM - SB which progressed to extreme bradycardia 30 bpm and complete AVB	LVH, MR, TR	Complete heart block with extreme bradycardia to 30 bpm preceded by SB	AVB Type III	ICU admission, atropine f.b. IV isoprenaline. LCM and bisoprolol dced.	No	No
2019, Ng [17]	48/F	None	Overdose toxicity	Widened QRS~118ms (baseline 88 ms)	NA	Widening of QRS complex	Wide QRS complexes	Sodium bicarb without relief, with supportive care	No	No
2020, Hsu [18]	73/M	CAD, CHF, HTN, Afib, HLD, ICH	Seizures	No formal EKG	NA	Bradycardia with unstable hemodynamic that progressed to PEA	PEA	Atropine f.b.1 round CPR with epinephrine f.b. TCP and isoproterenol	Yes	No
2020,Majmundar [19]	95/M	TIA, HTN	Seizures	Baseline- 1^O^ AVB (PR 270 ms). After LCM- 1^O^ AVB (PR 378 ms) LBBB, wide QRS~200msReturned to baseline after LCM dced	NA	Lengthening PR interval, new-onset LBBB, widened QRS and episodes of SB to 30 bpm and sinus pauses~ 3 sec noted	Sinus pause	Switching LCM to topiramate, external pacing, and 24 hours observation in CCU	No	No
2020, Stamm [20]	32/M	Baseline-SB with early repolarization. Occasional intermittent palpitations at baseline	Seizures	1^o ^AVB, J point elevation, and early repolarization, EKG returning to baseline, sinus brady with PR WNL. Mobitz I after IV LCM	NA	1^O ^AVB with progression to Mobitz I after switching from PO to IV LCM	Mobitz type I	LCM dced	No	No
2020, Eleftheriou [21]	38/F	HOCM, HTN, prolonged QTc	Seizures	VF	NA	An episode of VT f.b. 27 episodes of life-threatening VF after a 3^rd^ IV dose of 400 mg LCM	VT	Cardioversion @200J, NSR after LCM discontinuation	Yes	No
2020, Corbellini [22]	88/M	Obesity, HTN, HLD, mild MR and AR, G1DD	Seizures	NSR, 66 bpm with 1^o ^AVB at baseline, after IV LCM -afib with RVR to 140 bpm	G1DD mild MR, mild AR	New-onset Afib with RVR @140 bpm	Afib	Amiodarone infusion, LCM dced	No	No

Data was collected by four authors that included demographic data, cardiovascular (CV) risk factors, indication for LCM use, electrocardiography (EKG) findings, transthoracic echocardiography (TTE) findings, type of arrhythmias, cardiac arrest, and management when available as shown in Table 1.

Result

Table 2 summarizes the result of our systematic review of LCM-related arrhythmias. A total of 17 cases were identified with a median age of 48+/- 27.2, of whom 59% were females and 41% were males. Seizure/epilepsy was the indication for LCM use in all cases (100%) however 5.88% of the cases that were reported had LCM toxicity due to suicidal overdose. The prevalence of cardiovascular/arrhythmogenic risk factors was hypertension (HTN) in 52.9%, hyperlipidemia (HLD) in 17.6%, hypokalemia in 17.6%, and coronary artery disease (CAD) in 11.8%. Obesity, atrial fibrillation, transient ischemic attack (TIA), hypertrophic cardiomyopathy, prolonged QTc, history of mitral and aortic valve replacement, and multifocal atrial tachycardia were reported in 5.9% in each. Beta-blocker usage was reported in 23.5% and use of flecainide in 5.9%. Ventricular tachycardia was the most reported LCM related arrhythmia (29.4%), followed by new-onset atrial fibrillation (17.6%), complete heart block (17.6%), Mobitz type 1 atrioventricular block (11.8%), sinus pauses (11.8%), pulseless electrical activity (5.9%) and widening QRS complex (5.9%). Transthoracic echocardiography (TTE) was done in six patients, all of whom had normal ejection fractions. Temporary pacemaker placement was reported in 17.6% and permanent pacemaker placement in 5.9%. Cardiac arrest was reported in 35.29% and death was reported in 5.9% of the cases.

**Table 2:** Summary of the result of the systematic review of LCM related arrhythmias. Abbreviations: HTN-hypertension; HLD-hyperlipidemia; CAD-coronary artery disease; DM-diabetes mellitus; Afib-atrial fibrillation; TIA-transient ischemic attack; TTE-transthoracic echocardiography; EF-ejection fraction

Cases identified (n)		17
Age; n ± SD		Median 48 ± 27.2
Sex; n (%)		
	Males	7/17 (41%)
	Females	10/17 (59%)
Indication of LCM; n		
	Overdose toxicity	2
	Seizures/epilepsy	15
Cardiovascular risk factors; n (%)		
	HTN	9/17, (2.9%)
	HLD	3/17, (17.6%)
	Hypokalemia	3/17, (17.6%)
	CAD	2/17, (11.8%)
	DM	1/17, (5.9%)
	Obesity	1/17, (5.9%)
	Afib	1/17, (5.9%)
	TIA	1/17, (5.9%)
	History of aortic and mitral valve replacement	1/17, (5.9%)
	Hypertrophic cardiomyopathy	1/17, (5.9%)
	Prolonged QTc	1/17, (5.9%)
	Multifocal atrial tachycardia	1/17, (5.9%)
Patients on relevant medications; n, (%)		
	Beta-Blockers	4/17, (23.5%)
	Flecainide	1/17, (5.9%)
Reported arrhythmia; n, (%)		
	Ventricular tachycardia	5/17, (29.4%)
	New onset AFib	3/17, (17.6%)
	Complete heart block	3/17, (17.6%)
	Mobitz type I	2/17, (11.8%)
	Sinus pause	2/17, (11.8%)
	Pulseless electrical activity	1/17, (5.9%)
	Widening of QRS complex	1/17, (5.9%)
TTE reported; n, (%)		
	TTE mentioned in 6 articles- all had normal EF	6/6, (100%)
	No TTE data available	12/17, (70.5%)
Pacemaker; n, (%)		
	Temporary pacing	3/17, (17.6%)
	Permanent pacemaker	1/17, (5.9%)
Cardiac arrest; n, (%)		6/17, (35.29%)
Death; n, (%)		1/17, (5.9%)

Discussion

LCM is a novel antiepileptic agent used for add-on therapy in patients with partial and secondarily generalized seizures [2]. Intravenous LCM has been used for the treatment of status epilepticus [2]. Its main mechanism of action is the enhancement of the slow inactivation of voltage-gated sodium channels thus reducing the ability of neurons to sustain prolonged firing bursts [3]. Experimental studies and clinical trials suggest that LCM acts upon both neurons and the heart and may increase the risk of cardiac arrythmias [4]. The action potentials of most of the cardiac tissues, including the His-Purkinje system, are generated through voltage-gated sodium channels however that of AV node is through voltage-gated calcium channels [4, 7]. As seen with other anticonvulsant agents, dose-dependent inhibition of sodium channels produces infra-Hisian delays and conduction defects that could be one of the postulated mechanisms of cardiac arrhythmias with the use of LCM [2, 4]. It follows linear pharmacokinetics with a maximum concentration reached within 1-4 hours and a half-life of 13 hours [2]. Clinical trials [4, 23] have shown the association of LCM, dose-ranging from 200-600 mg/day, with cardiac conduction defects including atrial fibrillation and flutter. Few case reports suggesting first-degree AV block and third-degree AV blocks with LCM use have been retrieved from the literature [10] [24]. Traditional sodium channel blocking agents such as carbamazepine and phenytoin cause the fast inactivation of voltage-dependent sodium channels and may have potential synergistic action with LCM [12]. 

However, clinical trials [25] did not show any additional adverse effects with concomitant use of other sodium channel blockers. Several studies, including our systematic review, have documented cardiac arrhythmias with the use of LCM hence recommendations before the initiation may include baseline EKG, evaluation of cardiac risk factors, electrolytes levels, and adjusting the dose of LCM based on renal and hepatic functions.

## Conclusions

Arrhythmias associated with the use of LCM in seizures have been documented in several case reports and studies. LCM inhibits the cardiac sodium channel by enhancing slow inactivation in a concentration-dependent manner. The timing of several cardiac arrhythmias after initiation of LCM and their resolution after discontinuation suggests a possible pro-arrhythmic role of LCM as described in Table 1. Identification of any pre-existing cardiac/arrhythmogenic risk factors may prove to be beneficial before commencing treatment with LCM. One must also consider reviewing the home medications and their dosage to prevent any drug-drug interactions with the use of LCM. In our systematic review study, ventricular tachycardia was the most reported of LCM-related arrhythmia, and wide QRS complex was the least common one. Discontinuation of LCM was one of the common factors that helped to restore the sinus rhythm in most of the studied case reports. Comparative studies may be warranted for defining the role of a higher dosage of LCM~600 mg/day as compared to ~400mg/day in causing arrhythmias. Further research and clinical trials are needed to explore the etiopathogenesis and causative relationship between the use of LCM and arrhythmias. In addition, further investigations are needed to study the cardiac effects of LCM and its interactions with other antiepileptic drugs involving the inhibition of cardiac sodium channels. Electrocardiographic (ECG) testing before and during LCM therapy along with close monitoring may help to avoid cardiac arrhythmias
